# Study of risk factors for suicide attempts in patients with bipolar disorder

**DOI:** 10.1192/j.eurpsy.2021.531

**Published:** 2021-08-13

**Authors:** N. Smaoui, A. Guermazi, I. Lajmi, R. Feki, S. Omri, M. Maalej Bouali, J. Ben Thabet, L. Zouari, N. Charfi, M. Maalej

**Affiliations:** 1 Psychiatry C Department, Hedi chaker university hospital, sfax, Tunisia; 2 Department Of Psychiatry “c”, Hedi Chaker University Hospital, Sfax, Tunisia

**Keywords:** Suicide attempts, bipolar disorder, Risk factors

## Abstract

**Introduction:**

Bipolar disorder (BD) has the highest suicide attempt rate among psychiatric disorders. Many factors are associated with the risk of suicide attempt in BD, but the relation between them has still not been explicitly stated.

**Objectives:**

This study aimed to examine the clinical variables characterizing patients with BD with prior suicide attempt (PSA).

**Methods:**

This was a descriptive and analytical study, conducted over 3 months, involving 31 euthymic patients with BD, followed up in the outpatient psychiatry department of Hedi Chaker University Hospital in Sfax (Tunisia). General, clinical and therapeutic data were collected using a pre-established questionnaire. Quality of life (QOL) was assessed with the «36 item Short-Form Health Survey» (SF-36). Impulsivity was assessed with the Barratt Impulsiveness Scale (BIS-11).

**Results:**

The mean age was 47.25 years and the sex ratio was 1.6. Family history of suicide attempts was found in 25% of cases. Mean score of SF36 was 34 and high degree of impulsivity was noted in 62.5% of cases. The frequency of BD patients with PSA was 12.3% (N=8), with two of these (25%) having more than one PSA. Comorbid alcohol abuse (p=0.000), somatic illness (p=0.013), high degree of impulsivity (p=0.032), and impaired quality of live (p=0.003) were significantly more frequent in BD patients with PSA.

**Conclusions:**

We found several clinical variables associated with PSA in BD patients. Even though these retrospective findings did not address causality, they could be clinically relevant to better understanding suicidal behavior in BD and adopting proper strategies to prevent suicide in higher risk patients.

